# Welcome to the Intrinsic Capacity, Frailty and Sarcopenia Research Conference for Healthy Longevity (ICFSR 2026) in Washington, DC

**DOI:** 10.1016/j.tjfa.2026.100174

**Published:** 2026-07-13

**Authors:** Peter M. Abadir

**Affiliations:** Johns Hopkins University, Baltimore, MD, USA

It’s a real pleasure to welcome you to Washington, DC for the Intrinsic Capacity, Frailty and Sarcopenia Research Conference for Healthy Longevity (ICFSR 2026). I’m grateful you’re here, and proud that this year’s meeting is co-hosted by the Johns Hopkins Artificial Intelligence and Technology Collaboratory for Aging Research (AITC) and the Johns Hopkins Older Americans Independence Center (OAIC).

If there is one idea that keeps me grounded and excited in this work, it’s that aging is not a single pathway. Frailty is not one syndrome with one trajectory, and sarcopenia is not one story with one endpoint. Two people can share the same chronological age, similar diagnoses, even similar “frailty scores,” and still look completely different in function, symptoms, recovery, and goals. That heterogeneity can be frustrating when we design studies, build measures, or compare results across cohorts…but it’s also the point. It’s the lived reality of older adults, and it’s why simplistic models keep letting us down.

Part of what excites me about ICFSR’s evolving vision is that it invites us to hold several truths at once. Frailty has taught us how vulnerability emerges, often quietly, through multiple interacting systems. Sarcopenia reminds us that muscle is not just strength; it’s metabolism, immune function, mobility, participation, and dignity. And intrinsic capacity shifts the center of gravity toward what people can do and sustain: the composite of physical and mental capacities that underpins function over time.

These constructs are sometimes treated like competitors, different frameworks, different instruments, different “camps.” I think the next phase of the field is to map them together, intentionally. Intrinsic capacity, frailty, and resilience are not separate continents; they are different views of the same landscape. One emphasizes reserves, another emphasizes risk, another emphasizes recovery. If we can align language, measurement, and trajectories across these views, we’ll be closer to trial-ready outcomes that matter—and closer to interventions that are timely, not late.

There’s a proverb I come back to when definitions start to feel like the whole conversation: when a finger points to the moon, it’s easy to stare at the finger. Our instruments, subdomains, cut-points, and composite scores matter, but they are still the finger. The moon is what older adults tell us, again and again, they value most: maintaining function, autonomy, and quality of life. The goal isn’t to win a terminology debate; it’s to build a shared, usable framework that helps us detect early change, personalize risk, and intervene before dependency becomes inevitable.

That is also why this is such a demanding area of science. Aging research forces us to work across time, across systems, across environments, and across disciplines. It pushes us to reconcile biology with behavior, clinical care with population health, and mechanistic insight with scalable implementation. It asks us to be rigorous, but also humble, because variability is not noise to be discarded; it’s signal we haven’t fully learned to read.

This year’s program reflects that momentum. Across 8 symposia and round tables, 95 oral communications, and 240 poster presentations, we’ll focus on intrinsic capacity as a pre-clinical and clinical trial-ready framework; biomarkers of aging, frailty, sarcopenia, and healthy longevity for personalized approaches; geroscience-based therapies to enhance resilience and delay dependency; and artificial intelligence as a path toward scalable prediction and prevention of age-related decline. We also have the privilege of honoring Dr. John Beard for his pivotal contributions to intrinsic capacity and the ICOPE program, and learning from his keynote on mapping intrinsic capacity trajectories across the life course.

Finally, I want to acknowledge something that is rapidly reshaping how we work: AI, including emerging generative AI, and the broader ecosystem of sensors, imaging, digital phenotyping, and data platforms. These are not “add-ons.” They are becoming part of the infrastructure of aging research: helping us harmonize complex datasets, detect subtle changes earlier, model trajectories rather than snapshots, and build tools that can travel from specialized centers to real-world settings. Just as important, they bring new partners to the table—engineers, data scientists, designers, and industry collaborators, who share the same goal, even if they speak a different technical language. If we do this well, we will not only accelerate discovery; we will improve integration and usability in the places that matter most: clinics, communities, and homes.

Thank you for being part of ICFSR 2026. I hope these next days are full of thoughtful debate, practical collaboration, and new connections, so that together we keep our eyes on the moon: preserving function, extending healthspan, and making healthy longevity more attainable for more people.

